#  Inquérito *online* sobre os motivos para hesitação vacinal contra a COVID-19 em crianças e adolescentes do Brasil 

**DOI:** 10.1590/0102-311XPT159122

**Published:** 2023-10-13

**Authors:** Pétala Tuani Candido de Oliveira Salvador, Kisna Yasmin Andrade Alves, Katiuscia Roseli Silva de Carvalho, Marcio Fernandes Nehab, Karla Gonçalves Camacho, Adriana Teixeira Reis, Maria de Fátima Junqueira-Marinho, Dimitri Marques Abramov, Zina Maria Almeida de Azevedo, Margarida dos Santos Salú, Zilton Farias Meira de Vasconcelos, Saint Clair dos Santos Gomes, Orli Carvalho da Silva, Daniella Campelo Batalha Cox Moore

**Affiliations:** 1 Universidade Federal do Rio Grande do Norte, Natal, Brasil.; 2 Secretaria de Saúde Pública do Rio Grande do Norte, Natal, Brasil.; 3 Instituto Nacional da Saúde da Mulher, da Criança e do Adolescente Fernandes Figueira, Fundação Oswaldo Cruz, Rio de Janeiro, Brasil.; 4 Universidade Federal Fluminense, Niterói, Brasil.

**Keywords:** COVID-19, Vacinas, Vacinas contra COVID-19, Recusa de Vacinação, Movimento contra Vacinação, COVID-19, Vaccines, COVID-19 Vaccines, Vaccination Refusal, Anti-Vaccination Movement, COVID-19, Vacunas, Vacunas contra la COVID-19, Negativa a la Vacunación, Movimiento Anti-Vacunación

## Abstract

Objetiva-se desvelar os motivos para hesitação vacinal de pais e/ou responsáveis de crianças e adolescentes para prevenção da COVID-19. Trata-se de um estudo descritivo, de abordagem qualitativa, que busca analisar as respostas da pergunta aberta “por que você não vai vacinar, não vacinou ou está na dúvida em vacinar as crianças e os adolescentes sob sua responsabilidade para prevenção da COVID-19?”. A pesquisa incluiu indivíduos adultos, brasileiros, residentes no país, responsáveis por crianças e adolescentes menores de 18 anos. A coleta de dados aconteceu de forma eletrônica entre os meses de novembro e dezembro de 2021. As respostas foram organizadas e processadas com suporte do software Iramuteq. O *corpus* textual desta pesquisa foi composto pela resposta de 1.896 participantes, constituído por 87% de hesitantes (1.650) e 13% (246) de pais que têm intenção de vacinar, mas que esboçaram algumas dúvidas e considerações a respeito da vacinação de crianças e adolescentes. São motivos pelos quais pais e/ou responsáveis não vacinaram ou estão na dúvida em vacinar as crianças e os adolescentes sob sua responsabilidade para prevenção da COVID-19: receio em razão de a vacina estar em fase experimental e medo das reações adversas e dos efeitos a longo prazo. Já os motivos para ausência de intenção de vacinar decorrem dos entendimentos dos participantes de que a COVID-19 em crianças não é grave, os riscos da vacinação são maiores do que os benefícios e o direito de escolha em não vacinar.

## Abstract (en)

The objective is to unveil the reasons for vaccine hesitancy among parents and/or guardians of children and adolescents toward the prevention of COVID-19. This is a descriptive study, with a qualitative approach that seeks to analyze the answers to the open question “Why will you not vaccinate or have not vaccinated or are in doubt about vaccinating the children and adolescents under your responsibility, for the prevention of COVID-19?”. The research included adult individuals, Brazilians, living in the country, responsible for children and adolescents under 18 years of age. Data collection took place electronically in November and December 2021. The answers were organized and processed with the support of the software Iramuteq. The textual corpus of this research was composed of the response of 1,896 participants, consisting of 87% who were hesitant (1,650) and 13% (246) of parents who intend to vaccinate but who outlined some doubts and considerations about the vaccination of children and adolescents. These are reasons why parents and/or guardians have not vaccinated or are in doubt about vaccinating the children and adolescents under their responsibility for the prevention of COVID-19: fears about vaccination regarding the conception that the vaccine is in the experimental phase, fear of adverse reactions and long-term effects. The reasons for the lack of intention to vaccinate stem from the understanding of the participants that COVID-19 in children is not serious, the risks of vaccination are greater than the benefits, and the right of choice not to vaccinate.

## Resumen

El objetivo es revelar los motivos de la indecisión a las vacunas de padres y/o responsables de niños y adolescentes para la prevención de COVID-19. Se trata de un estudio descriptivo con enfoque cualitativo, que busca analizar las respuestas a la pregunta abierta “¿Por qué no va a vacunar o no vacunó o tiene dudas en vacunar a los niños y los adolescentes bajo su responsabilidad para la prevención del COVID-19?”. La investigación incluyó individuos adultos, brasileños, residentes en el país, responsables de niños y adolescentes menores de 18 años. La recolección de datos se realizó de forma electrónica entre los meses de noviembre y diciembre de 2021. Las respuestas se organizaron y procesaron con ayuda del software Iramuteq. El corpus textual de esta investigación fue compuesto por la respuesta de 1.896 participantes, siendo constituido por el 87% de indecisos (1.650) y el 13% (246) de padres que tienen la intención de vacunar, pero que esbozaron algunas dudas y consideraciones respecto a la vacunación de niños y adolescentes. Son motivos por los cuales los padres y/o responsables no vacunaron o están en duda en vacunar a los niños y adolescentes bajo su responsabilidad para prevención del COVID-19: temores con la vacunación en cuanto a la concepción de que la vacuna está en fase experimental, miedo a las reacciones adversas y los efectos a largo plazo. Los motivos de la ausencia de intención en vacunar se deben a que los participantes entienden que el COVID-19 en niños no es grave, que los riesgos de la vacunación son mayores que los beneficios y que tienen derecho a decidir no vacunarse.

## Introdução

Criada em 1796 por Edward Jenner, a vacina tem a capacidade de prevenir o retorno de doenças já erradicadas ou controladas e de diminuir consideravelmente a morte precoce da população ^1^. Mas foi somente a partir do século passado que a vacinação passou a ter maior destaque e a marcar a história da ciência devido aos impactos causados no ser humano: longevidade e saúde ^2^.

No Brasil, o Programa Nacional de Imunizações (PNI), criado em 1973, apresenta-se como uma política capaz de impactar o perfil de morbimortalidade da população brasileira, repercutindo em mudanças nos campos político, epidemiológico e social ^3^. Com os bons resultados apresentados por meio de ações de promoção e proteção da saúde, o PNI tem se destacado nacionalmente e internacionalmente, sendo considerado referência mundial pela Organização Pan-Americana da Saúde (OPAS) ^4^.

Em meio a tal cenário já consolidado, destaca-se, na atualidade, a COVID-19, que tem mostrado quão vulnerável é a população humana diante de doenças infecciosas emergentes, principalmente quando não existem tratamentos curativos e vacinas para promover a prevenção desse agravo ^5^. Na perspectiva de conter a pandemia do novo coronavírus, iniciou-se uma corrida contra o tempo, em que o objetivo principal era a criação de vacinas em curto espaço cronológico ^6^.

A vacinação contra a COVID-19 no território brasileiro iniciou no dia 17 de janeiro de 2021 com o uso de doses da vacina do laboratório Sinovac em parceria com o Instituto Butantan, que contemplou inicialmente os grupos de trabalhadores de saúde (inicialmente os que faziam parte da linha de frente dos serviços), idosos residentes em instituições de longa permanência, indivíduos com mais de 18 anos de idade com deficiência, pessoas que viviam em residências inclusivas e povos indígenas ^7^.

Conforme o *Plano Nacional de Operacionalização da Vacinação contra a COVID-19*, até o dia 1º de fevereiro de 2022, o Brasil já fazia uso de quatro vacinas autorizadas pela Agência Nacional de Vigilância Sanitária (Anvisa), duas delas com autorização para uso emergencial (Sinovac/Butantan e Janssen) e duas com registro definitivo (AstraZeneca/Fiocruz e Pfizer/Wyeth) ^4^.

Por sua vez, crianças e adolescentes na faixa etária de 12 a 17 anos tiveram sua vacinação contra a COVID-19 iniciada no dia 15 de setembro de 2021 ^7^. Já o público de crianças de 5 a 11 anos foi incluído na campanha de vacinação contra a COVID-19 no dia 5 de janeiro de 2022 por meio da *Nota Técnica nº 02/2022*, a qual destaca que para esse grupo o Ministério da Saúde rotula como “vacinação não obrigatória”. Em 14 de julho de 2022, a Anvisa aprovou a ampliação da vacinação de crianças de 3 a 5 anos contra a COVID-19 ^4^.

Sabe-se que a vacinação de crianças é capaz de protegê-las da COVID-19 na forma grave, assim como das complicações de curto e longo prazo ^8^. Em contraponto, o PNI tem enfrentado o desafio de alcançar as coberturas vacinais infantis. São vários os motivos relacionados a essa problemática, como a hesitação vacinal, que é uma das principais inquietações tanto para gestores como para pesquisadores brasileiros ^9^.

A recusa vacinal não é um problema recente, ela surgiu no fim do século XVII logo após a ocorrência da varíola. A palavra hesitação é mais utilizada nos dias atuais para nomear o processo de tomada de decisão em que os indivíduos: são motivados pela falta de confiança nas vacinas, nos profissionais de saúde e na sua eficácia; não apresentam entendimento sobre os riscos das doenças imunopreveníveis ou sobre a importância das vacinas; e/ou aqueles que por conveniência utilizam os motivos de falta de acesso ou indisponibilidade da vacina nos serviços de saúde para fortalecer os motivos para a recusa ^10^.

Outrossim, apesar de a vacinação contra a COVID-19 ter apresentado importante contribuição para o controle da pandemia, protegendo a população do adoecimento e principalmente prevenindo as ocorrências das formas mais graves da doença, a hesitação vacinal representa uma das principais barreiras para o progresso dessa ação. Dada a sua relevância, ressalta-se, ainda, que a hesitação vacinal foi considerada pela Organização Mundial da Saúde (OMS) ^11^ uma das dez ameaças à saúde pública e tem sido foco de estudos em nível mundial, como o Vaccine Confidence Project [Projeto de Confiança Vacinal].

Destarte, este estudo objetiva desvelar os motivos para hesitação vacinal de pais e/ou responsáveis de crianças e adolescentes para prevenção da COVID-19.

## Percurso metodológico

Trata-se de estudo descritivo, de abordagem qualitativa, que incluiu indivíduos adultos, brasileiros, residentes no país, responsáveis por crianças e adolescentes menores de 18 anos.

A coleta de dados aconteceu de forma eletrônica entre os meses de novembro e dezembro de 2021, a partir de instrumento de pesquisa construído na plataforma Google Forms (https://workspace.google.com/products/forms/). O formulário era composto por 35 questões, envolvendo dados demográficos e questões sobre COVID-19 e vacinas. Neste artigo, apresenta-se a análise da questão aberta, de resposta não obrigatória: por que você não vai vacinar, não vacinou ou está na dúvida em vacinar as crianças e os adolescentes sob sua responsabilidade para prevenção da COVID-19?

O estudo foi divulgado na página institucional de Internet do Instituto Nacional de Saúde da Mulher, da Criança e do Adolescente Fernandes Figueira (IFF; https://www.iff.fiocruz.br/), Fundação Oswaldo Cruz (Fiocruz), com o título *Estudo VacinaKids*, convidando para a participação (por meio de Facebook, Instagram e WhatsApp) e disponibilizando o *link* do formulário. Em uma estratégia de “bola de neve”, solicitou-se aos participantes convidar amigos, propagando exponencialmente o *link* nas redes sociais. Foi solicitado que responsáveis por crianças e adolescentes menores de 18 anos respondessem ao formulário, relatando sua pretensão futura de vacinação contra a COVID-19 quando a vacina estivesse autorizada pela Anvisa para sua idade.

Portanto, enquanto uma pesquisa de opinião pública em contexto *web*, o processo de amostragem se deu a partir da divulgação do *link* do instrumento de pesquisa nas mídias da instituição proponente e do incentivo aos participantes para sua propagação, sem uma definição prévia da amostra.

**Figura 1:**
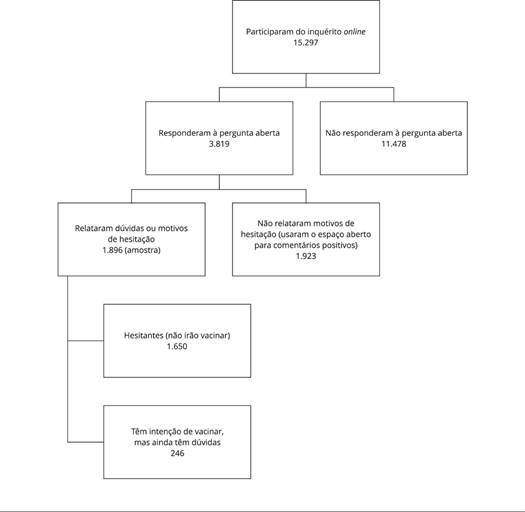
Fluxograma de composição da amostra.

Responderam ao inquérito *online* 15.297 indivíduos, e a análise quantitativa dos dados de caracterização e hesitação vacinal está publicada na íntegra ^12^. Destes, responderam à pergunta aberta 3.819 responsáveis. Foi necessário, todavia, excluir registros que não se referiam aos motivos para hesitação vacinal, uma vez que vários sujeitos utilizaram a questão aberta para realizar comentários positivos ou afirmar seu desejo de vacinar. Com isso, o *corpus* textual desta pesquisa foi composto pela resposta de 1.896 participantes, constituído por 87% de hesitantes (1.650) e 13% (246) de pais que têm intenção de vacinar, mas que esboçaram algumas dúvidas e considerações a respeito da vacinação de crianças e adolescentes (Figura 1).

Tais respostas foram organizadas e processadas com suporte do software Iramuteq (http://www.iramuteq.org/), o qual processa análises lexicais de dados textuais ao fornecer contextos e classes por meio do julgamento da semelhança de seus vocabulários, de maneira a contribuir para a compreensão do ambiente de sentido das palavras e, logo, indicar elementos das representações referentes ao objeto estudado ^13^.

A identificação dos sujeitos aconteceu a partir da sistematização de linhas de comando no corpus textual, compostas pelas seguintes variáveis: região do país; idade; sexo; escolaridade; se foi vacinado contra a COVID-19; medo de reações adversas; e se o filho teve COVID-19.

Foi utilizada a classificação hierárquica descendente (CHD) como método de tratamento dos dados, o que possibilitou identificar a frequência de cada palavra e sua conexão com as outras, além de auxiliar na análise do *corpus* textual ^13^. Consideraram-se 75% o valor de aproveitamento mínimo aceitável na análise lexicográfica dos segmentos de texto ^13^.

A CHD constitui método de tratamento de dados que visa obter classes de segmentos de texto que, ao mesmo tempo, apresentam vocabulário semelhante entre si e vocabulário diferente dos segmentos de texto das outras classes, a partir de uma análise lexicográfica que permite contextualizar o vocabulário típico de cada classe ^13^. Assim, as percepções dos sujeitos são agrupadas em classes com vocabulário semelhante, emergindo, dessa forma, as categorias de análise decorrentes das respostas dos participantes da pesquisa.

Ressalta-se que a interpretação e a análise dos dados tiveram embasamento da literatura atual sobre o objeto de estudo.

O estudo seguiu os princípios éticos e legais que regem a pesquisa científica com seres humanos e foi aprovado pelo Comitê de Ética em Pesquisa do IFF/Fiocruz (CAAE: 53087121.0.0000.5269, parecer nº 5.105.406, aprovado em 15 de novembro de 2021). O questionário foi preenchido somente após concordância com o consentimento informado.

## Resultados

Expuseram motivos de hesitação em vacinar crianças e adolescentes sob sua responsabilidade 1.896 sujeitos, o que corresponde a 12,2% do total de respondentes da pesquisa. O *corpus* textual composto pelas respostas dos participantes apresentou 64.472 ocorrências de palavras e 4.928 formas distintas, com aproveitamento de texto de 92,09% na análise da CHD.

**Figura 2:**
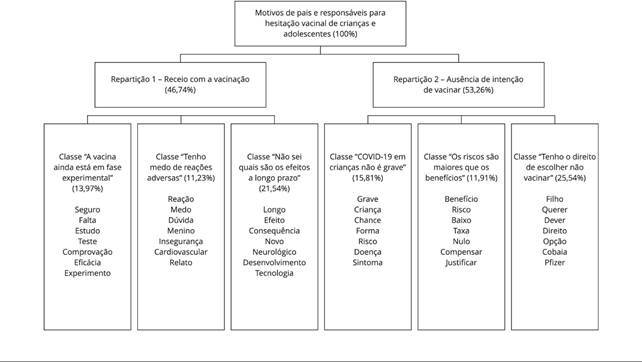
Dendrograma da análise das percepções dos participantes.

A análise lexicográfica agrupou os textos em duas repartições e seis classes. A Figura 2 apresenta a porcentagem de respostas e as palavras que tiveram destaque em cada classe.

A repartição 1 integrou um pouco menos da metade da percepção dos participantes (46,74%) e três classes que expressavam receios com a vacinação de crianças e adolescentes, perpassando a impressão de falta de mais estudos sobre segurança e eficácia e o medo quanto a reações adversas e efeitos de longo prazo.

A classe “A vacina ainda está em fase experimental” (13,97%) apresentou como palavras mais significativas “seguro”, “falta” e “estudo”. As respostas dessa classe revelam a concepção de que são necessários mais estudos nas faixas etárias das crianças, o que gera sentimento de insegurança nos pais em relação à vacinação de seus filhos, conforme observado nas falas a seguir:

“*Tenho medo de vacinar porque os estudos e testes ainda são muito recentes*” (homem, Região Sudeste).

“*Não vacinarei por tudo ainda ser incerto para as crianças. Com mais estudos e comprovações ficarei mais segura e tomarei minha decisão*” (homem, Região Nordeste).

As classes “Tenho medo de reações adversas” (11,23%) e “Não sei quais são os efeitos a longo prazo” (21,54%) também revelaram motivos de dúvidas dos pais quanto a vacinar seus filhos devido ao medo de possíveis efeitos que a vacinação contra a COVID-19 poderia ocasionar em crianças.

Na classe “Tenho medo de reações adversas”, as palavras “reação”, “relato”, “menino” e “cardiovascular” tiveram destaque e revelaram inseguranças específicas quanto a reações cardiovasculares devido a relatos que leram ou escutaram em alguma fonte, conforme demonstrado nas falas:

“*Tenho medo de reações adversas, principalmente eventos cardiovasculares*” (mulher, Região Nordeste).

“*Tenho medo de reações adversas cardiovasculares graves e permanentes*” (homem, Região Sudeste).

“*Tenho conhecimento de vários adolescentes com reações adversas*” (mulher, Região Centro-oeste).

“*Tenho medo de reações da vacina, em especial a Pfizer, em meninos, em razão de notícias que circulam sobre maiores chances de efeitos colaterais em meninos*” (mulher, Região Sudeste).

“*Não vacinarei devido à insegurança e ao medo de reações adversas a longo prazo*” (mulher, Região Norte).

Alguns participantes relataram o desejo de verificar primeiro como as crianças do convívio de seus filhos iriam reagir à vacinação para, após isso, tomar a decisão de vacinar suas crianças:

“*Gostaria de verificar quais reações possíveis na comunidade que convivo*” (mulher, Região Sudeste).

No que concerne à classe “Não sei quais são os efeitos a longo prazo”, ter Ensino Superior completo foi uma variável significativa (p < 0,005), com destaque para os vocábulos: “longo”, “efeito”, “neurológico” e “tecnologia”. Os discursos agrupados nessa classe revelam preocupação com os efeitos de longo prazo da vacinação contra a COVID-19, associados sobretudo ao fato de as crianças ainda estarem em desenvolvimento fisiológico, conforme pode ser observado nas falas:

“*Tenho medo de vacinar porque são vacinas novas* (...)*. Bebês e crianças estão em pleno desenvolvimento físico neurológico, não existe lógica em usar vacinas novas com pouco estudo e que ninguém tem como saber o que vai acontecer a longo prazo*” (mulher, Região Sudeste).

“*Tenho receio do impacto que a vacina possa ter a longo prazo no desenvolvimento neurológico dela*” (mulher, Região Sudeste).

Nessa classe, outro elemento muito enfatizado foi o medo das vacinas que utilizam tecnologias inovadoras em comparação às vacinas até então aplicadas em crianças. Foram vários os relatos da hesitação vacinal relacionada exclusivamente ao imunizante produzido pela Pfizer, com destaque para respostas com termos técnicos, o que pode estar relacionado ao fato de a escolaridade dos respondentes ter sido uma variável significativa nessa classe. Tais elementos estão exemplificados nas falas a seguir:

“*Tenho medo de vacinar porque desconheço os efeitos a longo prazo de uma vacina de RNA em células germinativas*” (homem, Região Sudeste).

“*Não vou vacinar, por ora, pois as vacinas aprovadas para crianças têm tecnologia de RNA para a qual não sabemos ainda as consequências a longo prazo*” (mulher, Região Sudeste).

“*Quero outro tipo de vacina, diferente da Pfizer*” (homem, Região Sudeste).

“*Não quero vacinar com a Pfizer*” (mulher, Região Centro-oeste).

Enquanto a repartição 1 apresentou um agrupamento de percepções relacionadas ao receio quanto à vacinação, na repartição 2 foi possível observar respostas de total negação quanto à vacinação em crianças e adolescentes, apoiadas em diferentes argumentos, conforme pode se observar nas classes apresentadas a partir de agora.

A classe “COVID-19 em crianças não é grave” integrou 15,81% das falas dos respondentes. Nela, duas variáveis de caracterização dos sujeitos foram elucidadas como significativas: o filho já ter tido COVID-19 (p < 0,05); e o pai ou responsável não ter se vacinado contra a COVID-19 (p < 0,0001). Destacaram-se os vocábulos “grave”, “criança”, “chance” e “risco” e a principal ideia sustentada nessa classe foi a concepção de que não é necessário imunizar crianças, já que não há chances de forma grave da doença nessas faixas etárias, discurso que pode ser observado nas falas a seguir:

“*Crianças não desenvolvem a doença de forma grave. Quem irá se responsabilizar pelos danos causados por esses produtos aprovados em caráter experimental?*” (mulher, Região Sudeste).

“*Os números da COVID-19, contágio em crianças e desfechos não se mostram críticos e aparentemente as crianças respondem bem ao processo infeccioso e, portanto, aplicar uma vacina que não impede o contágio, tampouco os desfechos graves, como tem ocorrido com adultos não faz sentido*” (mulher, Região Nordeste).

“*Crianças e adolescentes têm poucas chances de pegar COVID-19 e, caso peguem, a chance de ser grave é infinitamente menor que o risco da vacina*” (mulher, Região Sul).

De forma semelhante a tal compreensão acerca da vacinação em crianças como algo desnecessário, a classe “Os riscos são maiores que os benefícios” (11,91%) pautou-se em uma afirmação de que os riscos da vacinação em crianças superariam os da COVID-19, o que pode ser evidenciado no destaque dos vocábulos “risco”, “baixo” e “compensar” e nos discursos a seguir:

“*Estou vendo muitos relatos de efeitos colaterais que a mídia ou os órgãos responsáveis não estão relatando e nem orientando devidamente a população. E não sinto segurança nenhuma nessas vacinas, portanto, acho o risco de meus filhos terem COVID-19 grave muito baixo e acho o risco de efeito adverso da vacina muito maior*” (mulher, Região Sul).

“*Não vou vacinar porque a taxa de letalidade em crianças e adolescentes por COVID-19 é muito baixa e o risco de se usar imunizantes desenvolvidos em tão pouco tempo sem garantias de segurança, sobretudo no caso das vacinas genéticas, ainda é desconhecido, ou seja, a relação custo-benefício não compensa*” (mulher, Região Sudeste).

A classe “Tenho o direito de escolher não vacinar” agrupou mais de 1/4 da percepção dos respondentes, com destaque para as palavras “querer”, “opção”, “direito” e “cobaia”. Observaram-se nessa classe discursos enfáticos de hesitação vacinal, apoiados em uma concepção de liberdade de escolha quanto ao ato de vacinar:

“*Não sou cobaia humana de vacina, tampouco meus filhos, essas vacinas não imunizam ninguém e só causam danos*” (mulher, Região Sul).

“*Essas vacinas são experimentais e não sabemos plenamente o que elas contêm, temos direitos pela nossa constituição de optarmos pela nossa liberdade de escolha e de não arriscarmos a vida dos nossos filhos e netos*” (mulher, Região Sudeste).

“*O mínimo que deveria ser ofertado ao cidadão é o direito de escolher se realmente quer ou não se vacinar, sem contar que os efeitos colaterais e adversidades não são responsabilidade das indústrias, e sim dos governos que compram esses projetos de vacina. Os profissionais da saúde se corromperam e se prostituíram, não têm mais valores e nem princípio algum, nem mesmo empatia ou solidariedade com o próximo*” (homem, Região Centro-oeste).

## Discussão

A compreensão dos dados deste estudo demanda, inicialmente, o entendimento do cenário pandêmico no Brasil, que é constituído pelo fortalecimento do movimento antivacina e pelo negacionismo da pandemia e, consequentemente, pela presença de impactos negativos na implementação de ações para prevenção e controle da infecção pelo SARS-CoV-2 ^14^.

Transversalmente ao movimento antivacina e ao negacionismo da pandemia, há o negacionismo da ciência, em que “quem nega a gravidade da COVID-19 parte, muitas vezes, da negação dos discursos científicos”, e, portanto, a desqualificação dos cientistas ^15^. Reforça-se que, no meio desses movimentos, há uma narrativa orquestrada, ardilosa e alicerçada por interesses políticos e econômicos.

Posto isso, a repartição 1 do dendrograma (“Receios com a vacinação”) desvela que 46,74% dos participantes têm receio de vacinar seus filhos por diversos motivos/concepções: falta de estudos sobre segurança e eficácia da vacina e medo das reações adversas e dos efeitos de longo prazo. Além desses motivos identificados em nosso estudo, outros já foram pontuados: vacina nova, percepção de que a criança não corre risco de contrair COVID-19, recusa geral da vacina, falta de informações disponíveis/recomendadas sobre as vacinas ^16^.

Um estudo destaca que o desenvolvimento da vacina contra a COVID-19, que ocorreu em tempo recorde, com duração de meses - consequência do alto investimento pelos países -, associado à falta de conhecimento da população sobre o processo sistematizado e rigoroso da produção dos imunizantes, o uso inapropriado de termos epidemiológicos - e, por vezes, sem esclarecimentos à população, a exemplo do termo eficácia da vacina - e a socialização de informações incorretas e/ou falsas maximizaram os sentimentos de medo e insegurança ^17^.

Em coerência com esses achados, uma pesquisa realizada em Teresina (Piauí), no ano de 2020, em unidades básicas de saúde, demonstrou que os fatores decisivos para a não vacinação foram o descrédito na celeridade do processo de fabricação da vacina. Para os autores, é necessária a comunicação efetiva com a população, por meio de campanhas informativas relativas aos benefícios das vacinas aprovadas pela Anvisa ^18^.

Devido à urgência na produção de vacinas para o enfrentamento do SARS-CoV-2, passou-se a adotar estratégia do uso emergencial dos imunizantes. Em 18 de janeiro de 2021, a vacina contra a COVID-19 começou a ser aplicada no Brasil mediante aprovação emergencial do órgão regulador nacional, a Anvisa ^19^. Esse processo não implica uma autorização sem respaldo científico e requer a análise de dados referentes ao estudo de eficácia de fase 3, com seguimento mínimo de dois meses. Ainda, complementa-se a apreciação com os resultados das fases 1 e 2, os quais devem detalhar os eventos adversos e os fundamentos para acompanhamento de segurança a longo prazo ^20^. Denota-se que esses esclarecimentos não foram transmitidos de modo efetivo à população, o que potencializou o sentimento de insegurança.

No tocante ao “medo das reações adversas”, uma revisão sistemática da literatura ^21^, que objetivou avaliar a segurança das vacinas contra a COVID-19, revela a predominância de eventos adversos do tipo leve a moderado, como dor no local da aplicação do imunizante, edema, mialgia, febre, fadiga e cefaleia. Já os eventos adversos graves descritos nos estudos revisados não tinham relação com a vacina. Dessa forma, os autores concluem que as vacinas são seguras.

Outro ponto de discussão é a hesitação vacinal pela insegurança quanto às reações cardiovasculares pós-vacinação, em especial àquelas relacionadas às vacinas de mRNA. Há evidência de casos de mio/pericardite, especialmente após a segunda dose da vacinação. Contudo, são eventos raros e os benefícios superam os riscos das reações associadas ao imunizante ^22^. O Sistema de Notificação de Eventos Adversos de Vacinas dos Estados Unidos (VAERS; Vaccine Adverse Event Reporting System) reuniu dados de 8,7 milhões de doses aplicadas da vacina da Pfizer para prevenção da COVID-19 em crianças de 5 a 11 anos e mostrou a ocorrência de miocardite em apenas 11 casos, com evolução menos grave da observada com a infecção natural e sem ocorrência de óbito ^23^. Assim, observa-se que a ocorrência de miocardite pós-vacinal é um evento grave e autolimitado. No entanto, em decorrência das dúvidas geradas pelos movimentos antivacina e do negacionismo da ciência, enfatizou-se a persecução a essa temática.

No Brasil, os casos de eventos adversos pós-vacinação da COVID-19 ocorridos na faixa etária de 5 a 18 anos registrados pelos sistema de vigilância brasileiro no período de 18 de janeiro de 2021 a 18 de junho de 2022 mostram que após 37.205.093 doses de vacinas contra COVID-19 foram registradas 17.449 notificações de eventos adversos e apenas 546 (3,1%) de eventos adversos graves, sem nenhum óbito com relação causal com a vacina utilizada ^24^.

Ainda sobre as reações pós-vacinação, deve-se atentar que algumas condições biológicas, como a resposta imune robusta normal e esperada em grupos específicos - crianças e adolescentes, por exemplo -, e predisposições genéticas expliquem a presença de reações cardiovasculares em pessoas após recebimento de imunizante ^22^.

Com relação às vacinas produzidas pela tecnologia de mRNA, categorizadas no grupo de vacinas genéticas, a exemplo da Pfizer, o processo de produção de anticorpos não ocasiona alterações no organismo humano. A vacina tem parte do material genético do SARS-CoV-2 e, após sua administração, sensibiliza nosso sistema imunológico para produção de anticorpos, possibilitando uma boa resposta imune celular e humoral ^25^.

Além disso, o emprego de vacinas genéticas não é recente e sua utilização, há quase 30 anos, não se restringiu à prevenção e ao tratamento de doenças infecciosas, mas alcançou o cenário oncológico. Elas apresentam bom perfil de segurança, podem ser produzidas em larga escala, têm baixo custo e induzem o organismo a produzir títulos de anticorpos neutralizadores - capazes de bloquear a entrada do vírus nas células - maiores do que os presentes no soro humano, após recuperação da infecção ^26^^,^^27^.

Outro resultado identificado neste estudo foi a concepção de que a “COVID-19 em crianças não é grave”. De fato, a doença nesse público ocorre, na maioria das vezes, com quadros assintomáticos ou leves em comparação com adultos e idosos. Todavia, essa assertiva não denota afirmar que há inexistência de casos graves e óbitos ^28^.

Estima-se que nos Estados Unidos 40 milhões de crianças estão vulneráveis. Consequentemente, em caso de transmissão descontrolada entre esse grupo, pode-se vivenciar o cenário de vários óbitos e hospitalizações ^28^. É evidente que a busca por valores críticos de mortalidade por COVID-19 entre o público infantil, sem atentar para as nuances supracitadas, corresponde a uma análise incongruente aos determinantes do processo saúde e doença.

Ainda, ressalta-se que, segundo o *Boletim Epidemiológico nº 123* do Ministério da Saúde ^29^, até dia 23 de julho de 2022, foram registradas no Brasil 14.884 internações por síndrome respiratória aguda grave (SRAG) de pessoas com menos de 19 anos, com 638 óbitos nessa mesma faixa etária por COVID-19 em 2022. Vale ressaltar que na população entre 6 e 19 anos, faixa etária já contemplada para a vacinação, foram 4.492 internações e 259 óbitos por SRAG devido à COVID-19, letalidade de 5,7%. Esse número passa a soma de todos os óbitos para os quais há imunizante disponível e gratuito em todas as salas de vacinas espalhadas pelo país.

Na repartição 2 (“Ausência de intenção de vacinar”), a classe “Os riscos são maiores que os benefícios” revela a manipulação das informações a fim de subordinar um grupo da população para o não engajamento à vacinação. Essa constatação é pauta de discussões em todos os países e a desinformação, além de desencadear baixa adesão às campanhas, prolonga a pandemia, aumenta a suscetibilidade das crianças à infecção pelo SARS-CoV-2 e potencializa o sofrimento emocional decorrente da COVID-19, a saber: separação dos pais e amigos, hospitalizações e distanciamento social ^30^.

No tocante ao resultado da classe “Tenho o direito de escolher não vacinar”, as reflexões devem ser pautadas nos princípios éticos do direito individual e coletivo. Dessa forma, a não adesão à vacinação causa impactos na esfera individual, que variam entre adoecimento, hospitalização e óbito, mas sobretudo em âmbito coletivo, pois o sucesso da vacinação demanda a participação de 80% da população ^31^. Os grupos antivacina têm reforçado a ideia de que os pais têm direito de não vacinar seus filhos, concebendo direitos humanos como ausência de interferência, o que contrasta com o direito internacional que conceitua os direitos humanos positivamente ^32^. A saúde é definida pela OMS ^33^ como um estado de completo bem-estar físico, mental e social, e não apenas ausência de doença ou enfermidade. Ainda, consta no artigo 12 do Pacto Internacional sobre Direitos Econômicos, Sociais e Culturais a exigência de que os estados forneçam imunização contra as principais doenças que ocorrem na comunidade ^34^. Dessa forma, o acesso a imunizantes é um direito da criança a ter sua saúde garantida. Esse direito é ainda mais importante no Brasil, visto que a vulnerabilidade das condições socioeconômicas pode ser um fator de risco para a gravidade da COVID-19 ^35^. Tanto no Brasil como em outros países na América Latina e África, já foi observado que a gravidade da COVID-19 nas crianças é mais acentuada devido às comorbidades e aos sistemas de saúde precários dessas regiões ^36^.

Sabe-se que com a propagação de falsas informações e a negação da pandemia, principalmente por chefes do Poder Executivo que polarizam a população no contexto das definições estratégicas de prevenção e controle da COVID-19 por benefícios próprios e partidários, estimulam a sensação de que o direito individual é soberano e, portanto, o direito de escolha configura uma ação de liberdade. É preciso esclarecer que no combate ao SARS-CoV-2 a uniformidade é princípio mandatório. Afinal, a saúde é direito individual e coletivo e dever do Estado, não subordinado a grupos específicos.

Como limitação do estudo, destaca-se o possível viés de seleção da amostra, influenciado pelo acesso aos meios de divulgação do formulário no ambiente *online* e ao momento de coleta de dados, visto que, à época, a vacinação contra a COVID-19 ainda não havia sido iniciada no público infantil e, portanto, mapeou-se a intenção futura de vacinação. Além disso, a estratégia bola de neve acaba distribuindo o instrumento de coleta de dados para pessoas de um mesmo convívio social, com possível nível de crenças em saúde similar. Os resultados apresentados, portanto, devem ser analisados à luz de tais limitações.

## Considerações finais

São motivos pelos quais pais e/ou responsáveis não vacinaram ou estão na dúvida em vacinar as crianças e os adolescentes sob sua responsabilidade para prevenção da COVID-19: receio da vacinação devido à concepção de que a vacina está em fase experimental e medo das reações adversas e dos efeitos a longo prazo. Ao identificarmos fatores que contribuem para a hesitação vacinal em crianças, torna-se possível otimizar as estratégias para melhorar a aceitação da vacinação contra a COVID-19 pelos responsáveis.

Já os motivos para ausência de intenção de vacinar decorrem dos entendimentos dos participantes de que a COVID-19 em crianças não é grave, os riscos da vacinação são maiores do que os benefícios e o direito de escolha em não vacinar.

Destarte, é condição indispensável o delineamento de iniciativas - e consolidação das já existentes - que contribuam para a atenuação dos movimentos antivacina e para o fortalecimento da ciência, bem como para a divulgação efetiva dos seus feitos à saúde da população.
